# Risk of Severe Acute Exacerbation of Chronic HBV Infection Cancer Patients Who Underwent Chemotherapy and Did Not Receive Anti-Viral Prophylaxis

**DOI:** 10.1371/journal.pone.0132426

**Published:** 2015-08-14

**Authors:** Chih-An Shih, Wen-Chi Chen, Hsien-Chung Yu, Jin-Shiung Cheng, Kwok-Hung Lai, Jui-Ting Hsu, Hui-Chun Chen, Ping-I Hsu

**Affiliations:** 1 Division of Gastroenterology, Department of Internal Medicine, Kaohsiung Veterans General Hospital, Kaohsiung City, Taiwan; 2 Division of General Internal Medicine, Department of Internal Medicine, Kaohsiung Veterans General Hospital, Kaohsiung City, Taiwan; 3 National Yang-Ming University, Taipei City, Taiwan; 4 Department of Radiation Oncology, Kaohsiung Chang Gung Memorial Hospital, Kaohsiung City, Taiwan; Centers for Disease Control and Prevention, UNITED STATES

## Abstract

**Background:**

Reactivation of HBV replication with an increase in serum HBV DNA and alanine aminotransferase (ALT) activity has been reported in 20–50% of hepatitis B carriers undergoing cytotoxic chemotherapy for cancer treatment. Manifestation of HBV reactivation ranges from asymptomatic self-limiting hepatitis to severe progressive hepatic failure and fatal consequences.

**Aim:**

To investigate the risk of severe acute exacerbation of chronic HBV infection in HBsAg-positive cancer patients with solid tumors or hematological malignancies who underwent chemotherapy without antiviral prophylaxis.

**Methods:**

A retrospective review of charts was conducted for HBsAg-positive cancer patients in our institution who underwent chemotherapy and did not receive anti-viral prophylaxis between the periods of July 2007 to January 2013. We investigate the incidence of severe acute exacerbation of chronic HBV infection if these patients with a variety of solid tumors and hematological malignancies.

**Results:**

A total of 156 patients (hematological malignancies: 16; solid tumors: 140) were included. The incidence of severe acute HBV exacerbation in the patients with hematological malignancy was higher than that in solid tumors (25.0% [4/16] *vs* 4.3% [6/140]); *P* = 0.005). Additionally, patients receiving rituximab-based chemotherapy had higher acute exacerbation rate than those with non-rituximab-based chemotherapy (40.0% *vs* 4.1%, *P* = 0.001). Among the patients with solid tumors, the incidences of severe acute exacerbation of chronic HBV in hepatocellular carcinoma, colorectal cancer, lung cancer, breast cancer, gynecological cancer, urological tract cancer, head/neck cancer and other solid malignancies were 2.3%, 4.0%, 7.1%, 9.0%, 16.7%, 6.7%, 0% and 0%, respectively.

**Conclusion:**

Severe acute exacerbation of chronic HBV infection may occur in HBsAg-positive patients with a variety of solid tumors who received chemotherapy without adequate anti-viral prophylaxis. Hematological malignancy and rituximab-based chemotherapy are the risk factors related to severe acute exacerbation of chronic HBV infection in HBsAg-positive cancer patients undergoing chemotherapy.

## Introduction

Hepatitis B virus (HBV) infection is a global major health problem, with an estimate of 400 million chronic carriers of the HBV surface antigen (HBsAg) worldwide [1]. Chronic HBV infection is endemic in many parts of the world and in several populations, particularly in Asia, over 10% of the adult population are chronically infected with HBsAg-positive status [1–3]. Reactivation of HBV replication with an increase in serum HBV DNA and alanine aminotransferase (ALT) activity has been reported in 20–50% of hepatitis B carriers undergoing cytotoxic chemotherapy for cancer treatment without anti-viral prophylaxis [4–9]. A previous study has reported that the reactivation rate of HBV infection after chemotherapy was as high as 73% [10]. HBV reactivation has been reported frequently in patients diagnosed to have lymphoma or breast cancer and those who have received anticancer chemotherapy, and in this setting, the use of anthracyclines and corticosteroids as part of the chemotherapeutic combination and/or anti-emetic pre-medication were the factors shown to be associated with HBV reactivation [4,11]. Manifestation of HBV reactivation ranges from asymptomatic self-limiting hepatitis to severe progressive hepatic failure and fatal consequences [12–13].

Administering oral anti-HBV agents before chemotherapy is an effective means of reducing acute exacerbation of HBV infection and preventing fatal complications in patients with chronic HBV infection [14,15]. Both the American Association of Study in Liver Diseases (AASLD) and the European Association for the Study of Liver Disease (EASL) have recommended HBV testing before starting chemotherapy and administering oral anti-viral agents for HBsAg-positive patients [13,16]. However, the HBV infection testing rates before chemotherapy are extremely low, ranging from 14% to 31% [17–19]. A low HBV testing rate before chemotherapy is possibly owing to that chemotherapy is conducted by physicians from various hospital departments whose perceptions with respect to the importance of pre-chemotherapy HBV screening and HBV prophylaxis widely vary [20]. Currently, the exact severe acute exacerbation rates of chronic HBV infection in HBsAg-positive cancer patients with various solid tumors receiving chemotherapy have rarely been systemically assessed. In addition, most studies investigating flare-up of HBV infection in cancer patients receiving chemotherapy dealt with reactivation of HBV infection but not clinically significant acute exacerbation events [9].

In this study we aimed to investigate the risk of severe acute exacerbation of chronic HBV infection in HBsAg-positive cancer patients with solid tumors and hematological malignancies who underwent chemotherapy without antiviral prophylaxis in a HBV endemic area. The severe acute exacerbation rates of HBV infection in HBsAg-positive cancer patients with various solid tumors who underwent chemotherapy without HBV prophylaxis were systemically investigated.

## Materials and Methods

### Patients

We conducted a retrospective review of charts of newly diagnosed cancer patients who underwent systemic intravenous or oral chemotherapy at our institution between the periods of July 2007 to January 2013. This study was approved by Veteran General Hospital Kaohsiung (VGHKS) institutional review board, which waived informed consent requirement (VGHKS13-CT6-12) and the patient record was anonymized and de-identified prior to analysis. Patients with positive test of HBsAg at the onset of chemotherapy who did not receive anti-viral prophylaxis were included for the study. Exclusion criteria included (1) local chemotherapy with intracavity instillation of cytotoxic agents, (2) cancer treatment with small molecule kinase inhibitor targeted therapy (e.g., imatinib, sorafenib) or hormone therapy alone, (3) drug or alcohol related hepatitis, and (4) patients with positive results of serum anti-HCV antibody. This study was approved by our institutional review board, which waived informed consent requirement.

### Study Design

Patient charts were reviewed and data were recorded regarding age, gender, type of malignancy, tumor stage, chemotherapy agents, viral markers, HBV viral load and results of serum liver biochemical tests, creatinine level and prothrombin time from starting chemotherapy to 6 months after chemotherapy. Types of cancer were classified as hematological malignancy and solid tumor. Tumor stages were classified according to the AJCC TNM staging system. Type of chemotherapy was classified according to the presence of rituximab or not [14].

Because not all patients with HBV infection had data of HBV DNA level at baseline, the incidence of HBV reactivation could not be determined and was not included as outcome measure. Instead, main outcome measure was severe acute exacerbation of HBV infection. Severe acute exacerbation of HBV infection was defined as (1) serum alanine aminotransferase (ALT) increased beyond 10 times the upper limit of normal (ALT level ≧ 400 IU/L) during chemotherapy or 6 months following chemotherapy, (2) the presence of serum hepatitis B surface antigen at acute exacerbation, and (3) exclusion of liver injury due to superinfection or co-infection with hepatitis A, C, and D viruses, alcoholic liver disease, autoimmune hepatitis, drug hepatitis or major systemic events (e.g., shock, hypoxia, hemolytic anemia). The suspected severe acute exacerbation events were adjudicated by a hepatic injury review panel for the study according to the clinical courses, serological markers, and HBV DNA levels during acute events. Alcohol- or drug-induced liver injury was excluded by careful review of medical history and clinical course, and liver injury within 7 days following initiation of chemotherapy in the first cycle of cancer treatment was excluded for the possibility of chemotherapy agent-related hepatitis. Delayed chemotherapy treatment was defined as premature termination of treatment or delay of >8 days [9,21]. Patients with ALT ≧ 100 IU/L plus international normalized ratio (INR) ≧ 1.5, ascites, or encephalopathy were categorized as having liver decompensation [17]. Fatal HBV reactivation was defined as (1) fatal consequences due to complications of hepatic failure following severe acute exacerbation of HBV infection, and (2) exclusion of mortality caused by other major systemic diseases (e.g., acute myocardial infarct, cerebral vascular accident, and brain metastasis).

### Statistical Analysis

The compared data were analyzed using a Chi-square analysis or Fisher’s exact test. A value of *P* < 0.05 was considered significant. All statistical evaluations were performed using SPSS version 18.0 software.

## Results

**Fig 1:**
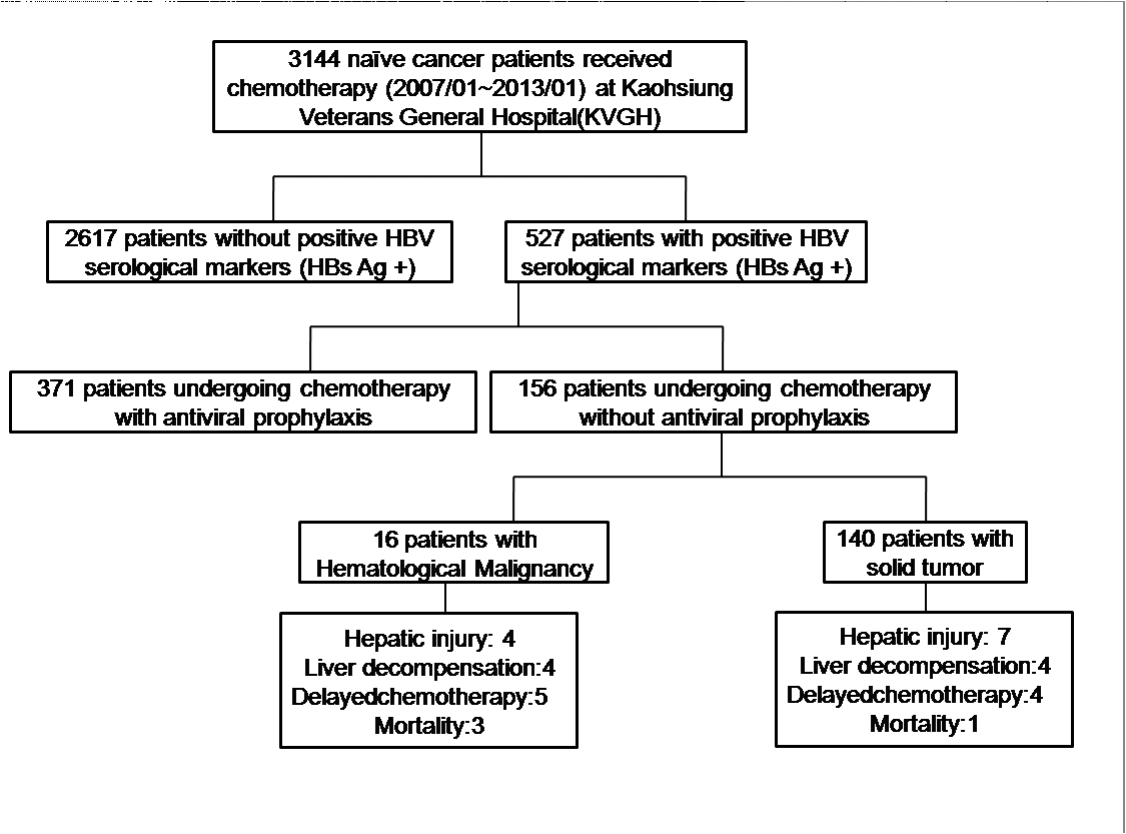
Patient disposition and clinical outcomes. A total of 156 patients were included for HBsAg-positive cancer patients in our institution who underwent chemotherapy and did not receive anti-viral prophylaxis between the periods of July 2007 to January 2013.

From July 2007 to January 2013, the charts of 3144 cancer patients undergoing chemotherapy were reviewed. A total of 156 HBsAg-positive cancer patients (hematological malignancies: 16; solid tumors: 140) were included (Fig 1). The clinical characteristics of the patients are summarized in Table 1. There were 107 (68.9%) males and 49 (31.4%) females. Most patients with hematological malignancies (81.3%) and non-HCC solid tumor had normal serum level of ALT at the onset of chemotherapy. In contrast, only 20.5% of the patients with HCC had normal ALT level before chemotherapy. None of patients with solid tumor received rituximab-containing chemotherapy. In the hematological malignancy group, 62.5% of the patients received chemotherapy containing rituximab (Table 1).

**Table 1:** Characteristics of the 156 HBsAg-positive cancer patients who received chemotherapy without antiviral prophylaxis.

Characteristics	Hematological Malignancy	Solid tumor	Total
	(n = 16)	(n = 140)	(n = 156)
**Age**			
< 60 yr	10(62.5)	75(53.6)	85(54.5)
≥ 60 yr	6(37.5)	65(46.4)	71(45.5)
**Sex**			
Men	10(62.5)	97(69.3)	107(68.9)
Woman	6(37.5)	43(30.7)	49(31.4)
**Pre-chemotherapy status**			
**ALT**			
Normal (<40U/L)	13(81.3)	71(50.7)	84(53.8)
Elevated	3(18.7)	69(49.3)	72(46.2)
**AST**			
Normal (<40U/L)	13(81.3)	79(56.4)	92(59.0)
Elevated	3(18.7)	61(43.6)	64(41.0)
**Total bilirubin**			
Normal (<1.6 mg/dL)	16(100)	132(94.3)	146(93.6)
Elevated	0(0)	8(5.7)	10(6.4)
**PT (INR)**			
Normal (<1.25)	16(100)	132(94.3)	148(94.9)
Elevated	0(0)	8(5.7)	8(5.1)
**Creatinine**			
Normal (<1.5 mg/dL)	16(100)	132(94.3)	148(94.9)
Elevated	0(0)	8(5.7)	8(5.1)
**Albumin**			
Normal(3.7~5.3 mg/dL)	12(75.0)	83(59.3)	95(60.9)
Elevated	4(25.0)	57(40.7)	61(39.1)
**HBe Ag**			
Positive	8(50.0)	56(40.0)	64 (41.0)
Negative	4(25.0)	8 (5.8)	12 (7.7)
No detected	4(25.0)	76 (54.2)	80 (51.3)
**Type chemotherapy**			
Rituximab (+)	10(62.5)	0(0.0)	10(6.4)
Rituximab (-)	6(37.5)	140(100.0)	146(93.6)

Table 2 showed the clinical outcomes of the HBsAg-positive cancer patients who received chemotherapy without antiviral prophylaxis. Severe acute exacerbation of HBV infection occurred in 25.0% (4/16) and 4.3% (6/140) of patients with hematological malignancy and solid tumor, respectively. Among the patients with solid tumors, the incidences of severe acute exacerbation of chronic HBV in HCC, colorectal cancer, lung cancer, breast cancer, gynecological cancer, urological tract cancer, head/neck cancer and other solid malignancies were 2.3%, 4.0%, 7.1%, 9.0%, 16.7%, 6.7%, 0% and 0%, respectively. Among the 16 patients with hematological malignancy, 25.0% developed liver decompensation due to severe acute exacerbation of HBV infection, and 18.7% died in fatal HBV reactivation. Additionally, delayed chemotherapy due to HBV flare up occurred in 31.3% of the patients.

**Table 2:** Severe acute exacerbation of HBV infection in HBsAg-positive cancer patients who received chemotherapy without antiviral prophylaxis.

Type of malignancy	No.	Severe acute exacerbation of HBV infection	Liver Decompensation	Delayed Chemotherapy	Mortality
**Hematological Malignancy** 16		4(25.0%)	4(25.0%)	5(31.3%)	3(18.7%)
Lymphoma	16	4(25.0%)	4(25.0%)	5(31.3%)	3(18.7%)
Leukemia	0	0(0%)	0(0%)	0(0%)	0(0%)
**Solid tumor**	140	6 (4.3%)	4 (2.9%)	3 (2.1%)	1 (0.7%)
HCC	44	1(2.3%)	2(4.5%)	1(2.3%)	0(0%)
Colorectal cancer	25	1(4.0%)	0(0%)	0(0%)	0(0%)
Lung cancer	14	1(7.1%)	1(7.1%)	2(14.2%)	0(0%)
Thymic cancer	0	0(0%)	0(0%)	0(0%)	0(0%)
Breast cancer	11	1(9.0%)	1(9.1%)	1(9.1%)	1(9.1%)
gynecological cancer	6	1(16.7%)	0(0%)	0(0%)	0(0%)
Esophageal cancer	5	0(0%)	0(0%)	0(0%)	0(0%)
Stomach cancer	2	0(0%)	0(0%)	0(0%)	0(0%)
Biliary tract cancer	0	0(0%)	0(0%)	0(0%)	0(0%)
Pancreas cancer	4	0(0%)	0(0%)	0(0%)	0(0%)
Head / Neck cancer	13	0(0%)	0(0%)	0(0%)	0(0%)
Urological tract cancer	15	1(6.7%)	0(0%)	0(0%)	0(0%)
Prostate cancer	0	0(0%)	0(0%)	0(0%)	0(0%)
Bone cancer	0	0(0%)	0(0%)	0(0%)	0(0%)
Musculoskeletal cancer	0	0(0%)	0(0%)	0(0%)	0(0%)
Others	2	0(0%)	0(0%)	0(0%)	0(0%)

Table 3 showed the univariate analysis of the clinical factors related to severe acute exacerbation of HBV infection in HBsAg-positive cancer patients who received chemotherapy without anti-viral prophylaxis. Type of malignancy and type of chemotherapy were associated with severe acute exacerbation of HBV infection. The incidences of severe acute HBV exacerbation in the patients with hematological malignancy and solid tumors were 25.0% and 4.3%, respectively. The former had higher incidence of severe acute HBV exacerbation than the latter (*P* = 0.005). Regarding the type of chemotherapy, rituximab-containing chemotherapy had a higher incidence of acute exacerbation than the non-rituximab-containing chemotherapy (*P* = 0.001). There were no significant differences with respect to age, sex, levels of ALT, AST, total bilirubin, albumin, INR, and creatinine.

**Table 3:** Univariate analysis for the clinical factors related to severe acute exacerbation of HBV infection in HBsAg-positive cancer patients who received chemotherapy without anti-viral prophylaxis.

	No. of Patients	Severe acute exacerbation of HBV infection	*P* Value
**Age**			0.348
< 60 yr	85	4(4.7%)	
≥ 60 yr	71	6(8.5%)	
**Sex**			0.431
Men	107	5(4.7%)	
Woman	49	5(10.2%)	
**Pre-chemotherapy status**			
**ALT (GPT)**			0.370
Normal (<40U/L)	84	4(4.8%)	
Elevated	72	6(8.3%)	
**AST (GOT)**			0.218
Normal (<40U/L)	92	4(4.3%)	
Elevated	64	6(9.4%)	
**Total bilirubin**			0.999
Normal (<1.6 mg/dL)	146	10(6.8%)	
Elevated	10	0(0%)	
**PTINR**			0.999
Normal (<1.25)	148	10(6.8%)	
Elevated	8	0(0%)	
**Creatinine**			0.999
Normal (<1.5 mg/dL)	148	10(6.8%)	
Elevated	8	0(0%)	
**Albumin**			0.952
Normal(3.7~5.3 mg/dL)	95	6(6.3%)	
Elevated	61	4(6.6%)	
**Type of tumor**			0.005
Hematological malignancy	16	4(25.0%)	
Solid tumor	140	6(4.3%)	
**Type of chemotherapy**			0.001
Rituximab (+)	10	4(40.0%)	
Rituximab (−)	146	6(4.1%)	

In this study, all the patients receiving rituximab-based chemotherapy had hematological malignancy. Additionally, the number of cases with acute exacerbation was small. Multiple regression analysis was therefore not performed to investigate the independent risk factors predicting severe acute exacerbation of chronic HBV infection.

Table 4 listed the clinical features of the ten patients who developed severe acute exacerbation of chronic HBV infection. In this study, 10 patients (lymphoma: n = 4; HCC: n = 1; lung cancer: n = 1; breast cancer: n = 1; colorectal cancer: n = 1; cervical cancer: n = 1; urological tract cancer: n = 1) developed severe acute exacerbation of HBV infection. All the severe acute exacerbation occurred after one or two cycles of chemotherapy. The median duration from initiation of chemotherapy to develop severe acute exacerbation of HBV infection was 75 days (range, 30–120 days). Fatal consequences due to severe acute exacerbation of HBV infection occurred in 4 patients.

**Table 4:** Clinical features of the ten patients who developed severe acute exacerbation of chronic HBV infection.

					Baseline				Peak value during chemotherapy							
Patient	Type of cancer	Chemotherapyagents	Gender	Age	HBsAg	HBsAb	ALT(IU/L)	HBV DNA	HBsAg	HBsAb	ALT(IU/L)	HBV DNA(IU/ml)	Time of HBV reactivation	Delayed of chemtherapy	Antiviral therapy	Mortality
1	Lymphoma	RCHOP	Male	67	positive	negative	17	Not done	positive	negative	3849	199880000	Two cycle later	No done	Telbivudine	+
2	Lymphoma	RCHOP	Female	79	positive	negative	21	Not done	positive	negative	2850	110986000	One cycle later	No done	Lamivudine+	+
															Entecavir	
3	Breast cancer	FAC	Female	58	positive	negative	49	Not done	positive	negative	3545	5121600	One cycle later	No done	Lamivudine+	+
															Entecavir	
4	Lymphoma	RCHOP	Female	54	positive	negative	17	Not done	positive	negative	3122	199880000	Two cycle later	No done	Lamivudine+	+
															adefovir	
5	Lung cancer	Etoposide + Cisplatin	Male	63	positive	negative	78	Not done	positive	negative	1306	4597800	One cycle later	60D	Entecavir	-
6	Urological cancer	MVEC	Female	65	positive	negative	23	Not done	positive	negative	495	Not done	One cycle later	60D	Lamivudine	-
7	HCC	HAIC	Male	70	positive	negative	21	Not done	positive	negative	597	2364	Two cycle later	30D	Telbivudine	-
8	Cervical cancer	Holoxan + Cisplatin	Female	60	positive	negative	20	Not done	positive	negative	614	551200	One cycle later	120D	Lamivudine	-
9	Colorectal cancer	FOLFIRI	Male	50	positive	negative	29	Not done	positive	negative	494	>1000000000	One cycle later	0D	Tenofovir	-
10	Lymphoma	RCHOP	Male	40	positive	negative	42	Not done	positive	negative	2233	352000000	One cycle later	0D	Entecavir	-

R-CHOP, rituximab, cyclophosphamide, vincristine and prednisolone; FAC, 5-fluorouracil, doxorubicin, cyclophosphamide; MVEC, methotrexate, vinblastine, epirubicin and cisplatinum; HAIC (hepatic arterial infusion chemotherapy), cisplatin, mitomycin, 5-fluorouracil and leucovorin; FOLFIRI, leucovorin, 5-fluorouracil, irinotecan

## Discussion

While HBV reactivation is a recognized complication in HBsAg-positive cancer patients undergoing chemotherapy, the exact frequency of severe acute exacerbation of HBV infection in these patients remains unclear. The current study showed the incidences of severe acute exacerbation of chronic HBV in HBsAg-positive patients with lymphoma, HCC, colorectal cancer, lung cancer, breast cancer, gynecological cancer, urological tract cancer, head/neck cancer and other solid malignancies were 25.0%, 2.3%, 4.0%, 7.1%, 9.0%, 16.7%, 20%, 0% and 0%, respectively. The mortality rate in the patients with severe acute exacerbation of HBV infection was 28.0% though all of them receiving antiviral therapy following HBV flare up. Our data suggest that pre-chemotherapy HBV screening and prophylaxis are indicated for patients with lymphoma and a variety of solid tumors including HCC, urological, gynecological, breast, colorectal, and lung cancers.

HBV reactivation during anticancer therapy has been well studied in lymphoma [10,22,23], and the majority of randomized controlled trials has focused on it [24,25]. High rates of HBV reactivation (from 24 to 88%) have been recognized in HBsAg-positive lymphoma patients undergoing rituximab plus steroid combination chemotherapy and hematopoietic stem-cell transplantation [22,26,27]. Increasingly, HBV reactivation is described in patients with solid tumors receiving chemotherapy, particularly in breast cancer patients receiving anthracycline-based regimens [27]. Yeo et al. reported that HBV reactivation rates in lymphoma, breast cancer, gastrointestinal cancer, head and neck cancer and lung cancer were 58%, 41%, 7%, 29% and 23%, respectively [9]. However, the clinical significance of HBV reactivation in HBsAg-positive cancer patients with solid tumors who receive chemotherapy remains unclear, and the exact frequencies of severe acute exacerbation of chronic HBV infection in HBsAg-positive cancer patients with solid tumors undergoing chemotherapy have not been systemically investigated. In this study, we demonstrated that the incidences of severe acute exacerbation of chronic HBV in HBsAg-positive patients with lung cancer, breast cancer, gynecological cancer, and urological tract cancer were above 5% (7.1%, 9.0%, 16.7%, and 6.7%, respectively). The novel data suggest that anti-HBV prophylaxis is not only indicated for lymphoma patients undergoing chemotherapy but also strongly recommended for those HBsAg-positive solid tumor patients with moderate risk (15–20%) of severe acute exacerbation.

Yeo et al. reported that pre-chemotherapy HBV DNA level, the use of steroids and a diagnosis of lymphoma or breast cancer were significant risk factors associated with HBV reactivation in cancer patients undergoing cytotoxic chemotherapy [9]. In this study, the incidence of severe acute HBV exacerbation in the patients with hematological malignancy was higher than that in solid tumors (25.0% *vs* 4.3%). Lok et al. [28] also reported hematological malignancy had higher incidence of severe acute HBV exacerbation than the solid malignant tumor (*P* = 0.005). Four (25%) out of HBsAg-positive diffuse large B-cell lymphoma (DLBCL) patients who underwent rituximab-containing chemotherapy without anti-viral prophylaxis. developed severe acute exacerbation of HBV infection. All the severe acute exacerbation occurred after one or two cycles of chemotherapy.

Rituximab is a chimeric mouse human anti-CD20 monoclonal antibody that can reduce B cell numbers and antibody levels. It is widely used as a single agent or in combination with chemotherapy in the management of CD20+ lymphomas, such as DLBCL and follicular lymphoma, and is a well known immunosuppressant associated with HBV reactivation. HBV reactivation may also occur in patients with resolved hepatitis B (HBsAg-negative/hepatitis B core antibody [anti-HBc]–positive) who receive rituximab. The incidence of HBV reactivation in patients with lymphoma and resolved hepatitis B after rituximab-based therapy ranges from 1.5% to 23.8% [29,30]. Therefore, screening for HBsAg & anti-HBc is strongly recommended for patients receiving with rituximab-containing chemotherapy, and antiviral prophylaxis should be considered in both HBsAg-positive cancer patients and those with resolved hepatitis B undergoing rituximab-containing chemotherapy.

Administering oral anti-HBV agents before chemotherapy is an effective means of reducing HBV reactivation and preventing fatal complications in patients with chronic HBV infection [14,15]. One of our recent studies also demonstrated that none (0%) of the 208 HBsAg-positive patients receiving HBV prophylaxis by oral anti-viral agents in our hospital from November 2009 to June 2013 developed severe acute exacerbation of HBV infection [31]. A meta-analysis by Lenna et al showed that patients given lamivudine prophylaxis reduced 87%, 70%, and 92% of HBV reactivation, reactivation related mortality, and chemotherapy disruptions, respectively [32]. Many clinical guidelines, including the American Association of Study in Liver Diseases (AASLD), European Association for the Study of Liver Disease (EASL) and United States Center for Disease Control and Prevention (CDC) therefore recommend the use of prophylactic antiviral agents for HBsAg-positive cancer patients undergoing cytotoxic chemotherapy [13,16,33].

Despite its contributions, this retrospective study has several limitations. Firstly, the numbers of HBsAg-positive patients in a variety of solid tumors were small. Secondly, it was a retrospective study. Therefore, some factors might influence physicians’ decisions to perform pre-chemotherapy screening and anti-viral prophylaxis or not. Thirdly, it was a single-institute study in an HBV endemic area. Nonetheless, it provided the incidence of severe acute exacerbation of HBV infection in hematological malignancy and a variety of solid tumors in HBsAg carriers who underwent chemotherapy without adequate HBV prophylaxis.

In conclusion, severe acute exacerbation of chronic HBV infection may occur in HBsAg-positive patients with a variety of solid tumors who received chemotherapy without adequate anti-viral prophylaxis. Hematological malignancy and rituximab-based chemotherapy are the risk factors related to severe acute exacerbation of chronic HBV infection in HBsAg-positive cancer patients undergoing chemotherapy.

## Supporting Information

S1 Data Underlying participant-level data are provided in a supporting information file. (XLS)
